# Perioperative fluid management in kidney transplantation: a black box

**DOI:** 10.1186/s13054-017-1928-2

**Published:** 2018-01-25

**Authors:** Maria Helena Calixto Fernandes, Thomas Schricker, Sheldon Magder, Roupen Hatzakorzian

**Affiliations:** 1 Department of Anesthesia, Royal Victoria Hospital, 1001 Decarie Blvd, Montreal, QC H4A 3J1 Canada; 2 Department of Critical Care Medicine, Royal Victoria Hospital, 1001 Decarie Blvd, Montreal, QC H4A 3J1 Canada

**Keywords:** Fluid management, Kidney transplantation, Delayed graft function, Perioperative volume infusion, Intravascular fluid assessment, Fluid responsiveness

## Abstract

The incidence of delayed graft function in patients undergoing kidney transplantation remains significant. Optimal fluid therapy has been shown to decrease delayed graft function after renal transplantation. Traditionally, the perioperative volume infusion regimen in this patient population has been guided by central venous pressure as an estimation of the patient’s volume status and mean arterial pressure, but this is based on sparse evidence from mostly retrospective observational studies. Excessive volume infusion to the point of no further fluid responsiveness can damage the endothelial glycocalyx and is no longer considered to be the best approach. However, achievement of adequate flow to maintain sufficient tissue perfusion without maximization of cardiac filling remains a challenge. Novel minimally invasive technologies seem to reliably assess volume responsiveness, heart function and perfusion adequacy. Prospective comparative clinical studies are required to better understand the use of dynamic analyses of flow parameters for adequate fluid management in kidney transplant recipients. We review perioperative fluid assessment techniques and discuss conventional and novel monitoring strategies in the kidney transplant recipient.

## Background

Despite improvements in outcomes for patients undergoing renal transplantation, delayed graft function (DGF) remains a significant complication and is a predictor of the subsequent clinical course [1]. Delayed graft function is associated with decreased graft and patient survival, impaired long-term function and increased acute rejection [2, 3]. Optimized perioperative hemodynamic management is effective for prevention of DGF [4, 5], but optimal fluid therapy remains a challenge.

The kidneys receive approximately 25% of the body’s total cardiac output (CO) and are essential for maintaining tonicity of body fluids and for adjusting extracellular volume. Patients with renal failure often have electrolyte imbalances and tend to oscillate between hypovolemia and hypervolemia [6]. This results in a very narrow margin of safety for intravenous fluid resuscitation and maintenance. Patients undergoing kidney transplantation are at risk of developing DGF, acute kidney injury (AKI) and fluid overload. Hypovolemia can lead to further kidney injury, but excessive fluid therapy can result in pulmonary edema. Optimal fluid management is essential to reduce perioperative complications.

Central venous pressure (CVP)-guided volume infusion is the traditional approach in renal transplantation [7, 8] and involves intraoperative infusion of large volumes of fluid. Maximal volume infusion to the point of no further fluid responsiveness has long been considered the best approach [9–11], but this can lead to excess fluid which can damage the endothelial glycocalyx and lead to a fluid shift into the interstitial space [12]. Furthermore, in this range, CO cannot be modified when needed by changes in cardiac filling pressure alone.

Previous studies on the assessment of intravascular volume and optimization of CO and renal blood flow have indicated that conventional monitoring provides insufficient data for adequate fluid management [13–16]. However, meta-analyses of randomized controlled trials indicate that approaches which include measurement of CO and calculate oxygen delivery to guide intravenous fluid replacement are associated with decreased mortality and postoperative complications [17–19].

In this article, we review perioperative fluid assessment techniques and discuss conventional and novel monitoring strategies in this challenging patient population.

## Main text

### Delayed graft function

The definition of DGF is not consistent in the literature. At least 18 different heterogeneous criteria were identified in a systematic review [20]. Delayed graft function is most commonly used to describe the failure of the transplanted kidney to function promptly after transplantation, leading to dialysis within 1 week after transplant [20, 21].

The reported incidence of DGF varies from 2 to 70% in deceased kidney transplants [22, 23], not surprising given the wide range of definition. Outcomes are best in living donor transplantation, in which rates are 4–10% [23]. Despite improvement in transplantation techniques, the incidence of DGF has not decreased. A possible explanation may be the increasing number of grafts from expanded criteria donors and nonheart-beating donations [23, 24].

The underlying pathophysiology of DGF can be donor, recipient or surgeon related and can be due to hemodynamic (ischemic–reperfusion) or immunological (particularly T-lymphocyte) processes. A key factor is occult imbalance between oxygen delivery and consumption in the graft [25].

Acute tubular necrosis (ATN) is the most common cause of DGF. Acute tubular necrosis can be already present at the time of organ procurement if the deceased donor’s kidney is injured. Prolonged warm and cold ischemia time, as well as the manner of preservation, also can lead to ATN [23]. Acute tubular necrosis of the transplanted kidney can be diagnosed initially by nuclear renal scan which shows gradual uptake but minimal or no excretion of the tracer [26]. The hemodynamic status of the recipient during renal transplantation, the time following kidney implantation and completion of the anastomosis, and reperfusion injury all can effect graft perfusion [27] and the development of DGF [4, 5, 28]. Furthermore, the transplanted kidney is denervated and lacks neurogenic regulation of renal blood flow [29]. The vasodilation caused by mediators that accumulate during the ischemia period, and the increase in vascular permeability, might contribute to the invariable decline in CVP, from the operating room to the postanesthesia care unit [30]. Continuous monitoring is therefore highly recommended in postoperative care.

**Fig. 1:**
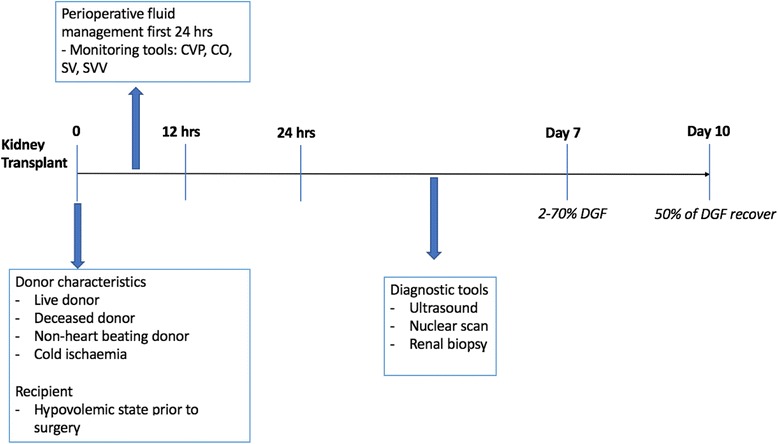
Kidney transplantation and DGF: timeline for risk factors and interventions. DGF delayed graft function, CVP central venous pressure, CO cardiac output, SV stoke volume, SVV stroke volume variation

Several epidemiological studies in different patient populations have confirmed an association between stages of AKI and short-term and long-term outcomes [31–33] as well as the independent association of AKI with higher mortality risk [34]. This is likely also true in kidney transplantation. Once the donated kidney has reached the implantation unit, donor and graft characteristics are no longer modifiable, and hemodynamic management becomes an adjustable portion of the process (Fig. 1).

In summary, DGF is predominantly caused by ATN. Adequate perfusion of the transplanted kidney is required, but to date no specific therapeutic intervention has reduced the incidence of ATN and recovery [35]. The role of health care professionals involved in perioperative fluid management of kidney transplantation is to identify the perfect balance of fluid therapy.

### Perioperative intravascular fluid assessment

The assessment of a patient’s intravascular volume for achievement of appropriate fluid management is a major challenge for the clinician and has been shown to improve postoperative outcomes [12]. Understanding of the following four definitions is essential in perioperative care:

1. Euvolemia: a state in which the vascular volume is adequate for filling of the heart and maintenance of an appropriate CO to generate the appropriate oxygen supply for tissue needs.
2. Fluid responsiveness: a state in which an increase in vascular volume increases the stroke volume (SV) and CO. The notion of fluid responsiveness does not necessarily mean that the patient will benefit from or need fluid therapy. Parameters of perfusion should guide the decision to assess the fluid response rather than responsiveness in a compromised patient.
3. Fluid overload: a state in which excessive accumulation of fluid in the vascular system is caused by excessive parenteral infusion or deficiencies in cardiovascular or renal fluid volume regulation.
4. Hypovolemia: a state in which effective circulatory volume is decreased and there is insufficient oxygen delivery to tissues, possibly resulting in organ dysfunction.

Despite a clear definition, there is no gold standard for the diagnosis and treatment of hypovolemia. Both acute and chronic conditions interact with fluid homeostasis, and medical history must always be considered. Progressive loss of renal function results in various adaptive and compensatory changes to maintain homeostasis with glomerular filtration rates below 10 ml/min, and these abnormalities typically have clinical consequences. A patient with end-stage renal disease has a disturbed acid–base and electrolyte balance and compromised hemodynamic autoregulation. Inflammatory mediators can alter renal hemodynamics and cause glomerular, tubular and interstitial damage, if uncontrolled [36].

In addition to the uncertainty associated with the patient’s volume state, appropriate targets remain unclear. To determine which monitoring method provides the most accurate information in the context of kidney transplantation, and which target to rely on, we will review previously implemented methods, their advantages and disadvantages, and their relative utility in predicting fluid responsiveness and tissue perfusion. Methods range from conventional techniques such as clinical history and physical examination, measurement of blood pressure (BP), heart rate (HR), urine output (UOP), CVP and pulmonary artery pressure (PAP), to novel technologies such as echocardiography, cardiorespiratory interactions and dynamic analyses of flow parameters.

### Conventional monitoring and targets

Clinical experience and use of traditional parameters such as BP, HR, UOP, CVP [37] and PAP to guide perioperative fluid therapy in kidney transplantation have been shown to be unreliable.

Hypovolemia in the perioperative setting often can be misdiagnosed clinically [38] due to a lack of obvious fluid loss in the context of altered systemic vascular resistance associated with capillary leak. An observational study in healthy volunteers during progressive hemorrhage showed that blood loss of 25% can appear with minimal clinical impacts on BP and HR, despite worsening microcirculatory flow [39]. Hypotension is not present in all patients in shock. If the physician waits for hypotension to start treating hypovolemia, tissue hypoxia will already be present [40].

In the case of hypovolemia and hypoperfusion, physiological compensatory mechanisms can maintain effective vascular volume and perfusion by increasing the vascular retention of an infused volume and decreasing vascular capacitance. Plasma levels of renin and aldosterone increase and atrial natriuretic factor decreases, followed by arterioconstriction which decreases precapillary hydrostatic pressure and filtration [41, 42]. A 30–50% reduction in glomerular filtration, and an increase in fluid reabsorption from the interstitial space, can also help avoid tissue edema [42, 43]. These compensatory mechanisms are highly compromised in end-stage renal disease and in the postischemic kidney.

Blood pressure is the most commonly used vital parameter for organ perfusion, and functions as an indirect measurement of renal perfusion in clinical practice. Although classic physiological studies suggest that autoregulation of renal blood flow is maintained at lower levels of mean arterial pressure (MAP 50–60 mmHg) [44, 45], retrospective data suggest that intraoperative MAP < 55 mmHg is associated with AKI and myocardial injury [46]. In a prospective randomized controlled trial in which MAP targets of 65–70 mmHg were compared to MAP targets of 80–85 mmHg in septic patients, there were no significant differences in mortality [47]. Renal replacement therapy was less commonly required in patients with chronic hypertension whose MAP was maintained at values of ≥ 80 mmHg [47]. However, raising MAP by increasing the systemic vascular resistance does not necessarily improve flow and potentially also not renal perfusion.

Given the expected large oscillations in hemodynamic parameters during kidney transplantation, invasive arterial BP monitoring is commonly used. In a recent study of the biochemical outcomes of renal transplant recipients, no difference was observed in the creatinine levels of patients with a MAP between 95 and 131 mmHg, but MAP was continuously maintained at > 95 mmHg via the judicious use of fluids and dopamine [27]. Consistent with other studies, Campos et al. [4] observed that MAP < 93 mmHg is associated with poor graft function. In an earlier study, Tóth et al. [11] observed stable creatinine levels in patients with a MAP of 80–100 mmHg, but an increase in creatinine in patients with MAP < 80 mmHg. However, these associations do not imply that raising pressure would restore function. The optimal measurement to evaluate renal microcirculation is unclear, as well as it being unknown which individual MAP could prevent AKI during different medical conditions.

Earlier studies have indicated that the magnitude of UO is one of the most clinically used predictors for graft function immediately after reperfusion [48] and proposed the use of CVP and PAP values to predict diuresis [8, 10]. Although aggressive volume expansion to a target CVP of 10–15 mmHg [49] and PAP > 20 mmHg [10] at the time of reperfusion were associated with improved renal blood flow and better graft outcomes, targeting these values was not recommended by other authors. A twofold greater risk of kidney dysfunction was observed with CVP ≥ 11 mmHg [4]. Overinfusion can lead to tissue edema with decreased tissue oxygenation [50] and subjects vulnerable patients to complications such as pulmonary edema, infections, myocardial ischemia, ileus, compromised renal flow, renal injury and even increased mortality [4, 12, 51–53].

A decrease in CVP in the post-transplant period is observed in a majority of kidney recipients, independent of their intravascular fluid status [30], but this does not mean the patient needs fluid. The etiology is unclear, but might be related to increased vascular permeability or the release of vasodilator mediators during the ischemic period. An increase in vascular capacitance also could be a factor [54]. Overemphasis on CVP as a goal can lead to excess infusion of fluid and increased capillary leakage, which can promote further fluid administration.

Use of pulmonary artery catheters has fallen out of favor due to concerns about complications such as pulmonary embolism among high-risk patients [55], pulmonary artery rupture [56] and cardiac perforation [57], and a frequent misinterpretation of the waveforms [58].

Static cardiac filling pressures such as CVP and PAP correlate poorly with the intravascular volume and are notoriously unreliable in predicting accurate fluid responsiveness compared to dynamic parameters [59, 60]. In a recent meta-analysis of the correlation between CVP and changes in cardiac performance, Marik and Cavallazzi [15] showed that only 57% of high-risk patients in the intensive care unit and operating room who appeared to be hypovolemic based on CVP were fluid responders, whereas the other half were unnecessarily loaded with fluids. However, a high CVP could potentially compromise renal flow, particularly when the arterial pressure is low.

Thus, the CVP should not be used alone to guide fluid responsiveness [15] and should not be a target for clinical decisions regarding fluid management [61]. Although the conventional parameters discussed previously are relevant to the hemodynamic assessment, none are good predictors of fluid responsiveness [59], which has prompted a search for alternative methods. There currently is increasing use of measurement of CO and SV with the aid of less invasive and easier to use devices. Use of these novel technologies to guide therapy is becoming vital in modern medical practice.

### Physiological background for a novel approach to fluid administration

The primary goal in the perioperative setting is to avoid tissue hypoxia, the major cause of organ dysfunction. Traditional indicators can be normal in the presence of tissue hypoxia and cannot be used to predict an imbalance between oxygen demand and consumption, particularly if they are not interpreted in the context of perfusion markers such as CO, lactates and central venous saturation [13–15, 61].

Sufficient oxygenation and tissue perfusion are crucial for all metabolic needs of cells and are based on hemoglobin concentration, arterial oxygen content and CO. Therefore, the percent change in any one of these three variables equally alters oxygen delivery, with CO usually having the largest changes [62]. As CO is the product of SV and HR, it can also be influenced by changes in preload, contractility and afterload. A mismatch between tissue needs and oxygen delivery leads to tachycardia and hypotension in most patients. Thus, an increase in preload with fluid therapy is only one part of treatment. An increase in contractility with inotropes remains the only therapeutic option when cardiac function is volume limited [62].

According to Frank–Starling’s law of the heart, administering fluids can increase the preload and thereby increase SV and CO. Under physiological conditions, both ventricles work on the ascending portion of the Frank–Starling curve, and afterload, contractility and HR are assumed to remain constant during the volume infusion [63]. This mechanism represents the functional preload reserve for the heart under stress conditions. If the patient is a fluid responder, this mechanism translates to a shift to the right of the venous return curve relative to the cardiac function curve and intersects it on the steep part of the cardiac function at a higher value [64]. A patient is considered a fluid responder when SV increases by at least 10–15% in response to volume expansion [65]. However, when the venous return intersects the flat part of the cardiac function curve there is no increase in CO and there even can be a decrease in ventricular performance and an increase in tissue edema and consequent tissue dysoxia. In nonresponders, CO only can rise by an increase in contractility or HR. The change in CVP in response to a fluid bolus can help interpret what happened with the fluid bolus when used in combination with the CO response.

Among the many devices and monitors currently available for measuring cardiac function and output, we will review the intraoperative use of transesophageal echocardiography (TEE) and emphasize the utility of noninvasive devices that calculate reliable trends in CO and dynamic indices based on simple physiology.

### Transesophageal echocardiography

Transesophageal echocardiography can be a useful diagnostic tool for evaluating right and left heart function, potential outflow tract obstruction and the presence of a pericardial effusion. It also can be used to obtain Doppler measurements of CO. However, TEE is operator dependent, and errors in the measurement of the diameter of the left ventricular outflow tract skew results. Presence of an irregular HR also affects the final calculation of CO.

Use of intraoperative TEE on renal allograft outcomes has only been evaluated in one study, in which living donor renal transplants were examined. Guiding fluid therapy in 110 patients by corrected flow time obtained with continuous transesophageal Doppler (TED) monitoring did not improve immediate graft function compared to fluid administration guided by CVP. However, significantly less fluid was used in the TED-monitored group, and the incidence of postoperative complications related to fluid overload was reduced [66].

Transesophageal eechocardiography does not permit continuous monitoring for instantaneous evaluation of fluid responsiveness. Transesophageal echocardiography is also time consuming and expensive, and requires intensive operator training. Careful attention must be given to the limitations of the assessment of CO by TEE, which may be beyond the level of expertise of a physician with only moderate experience. New studies with more robust data are required to support the use of TEE for fluid management in this patient population.

### Noninvasive dynamic CO technology

Cardiac output alone can be a poor predictor of fluid responsiveness, although its response to a preload-modifying maneuver such as mechanical ventilation can provide useful information about fluid responsiveness without the administration of fluid [67].

Currently there are several noninvasive CO monitoring technologies including pulse contour analysis (PCA), pulse wave transit time, thoracic electrical bioimpedance/bioreactance and CO_2_ rebreathing [68]. We will focus on parameters derived from technology using PCA to calculate CO. The SV is derived from the arterial waveform signal using the area under the systolic part of the arterial pressure curve. There are few devices using PCA technology: FloTrac/Vigileo/EV1000 system (Edwards Lifesciences, Irvine, CA, USA), LiDCOrapid (LiDCO, Cambridge, UK), MostCare (Vygon, Vytech, Padua, Italy) and ProAQT/PulsioFlex (Pulsion/MAQUET, Rastatt, Germany). Although the absolute CO values may not be as accurate as those derived by pulmonary artery catheter, these devices are very useful in providing acceptable trending abilities to follow changes in CO and SV. More studies are warranted to determine the effectiveness of these devices in improving patient outcomes [69].

**Fig. 2:**
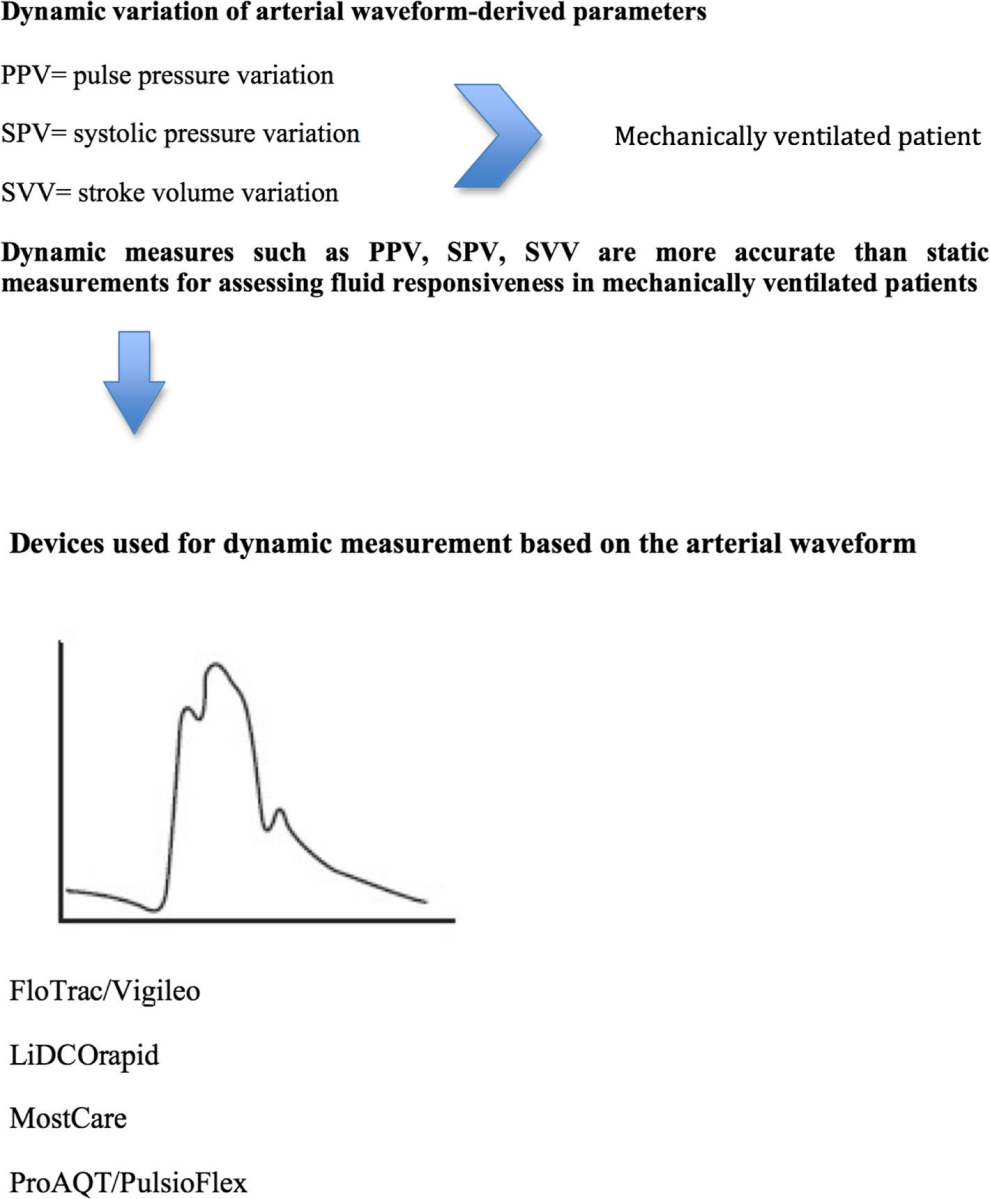
Dynamic parameters derived from the arterial waveform and available technologies

Dynamic variation of arterial waveform-derived parameters (i.e., systolic pressure variation (SPV), pulse pressure variation (PPV) and stroke volume variation (SVV)) in mechanically ventilated patients provides a precise indication of fluid responsiveness, particularly when compared to static indices [59]. However, SPV, PPV and SVV only can predict variations in the stroke volume index and cardiac index when the patient receives controlled ventilation and has no spontaneous respiratory efforts (Fig. 2). The percentages by which both parameters are likely to increase can also be predicted.

**Fig. 3:**
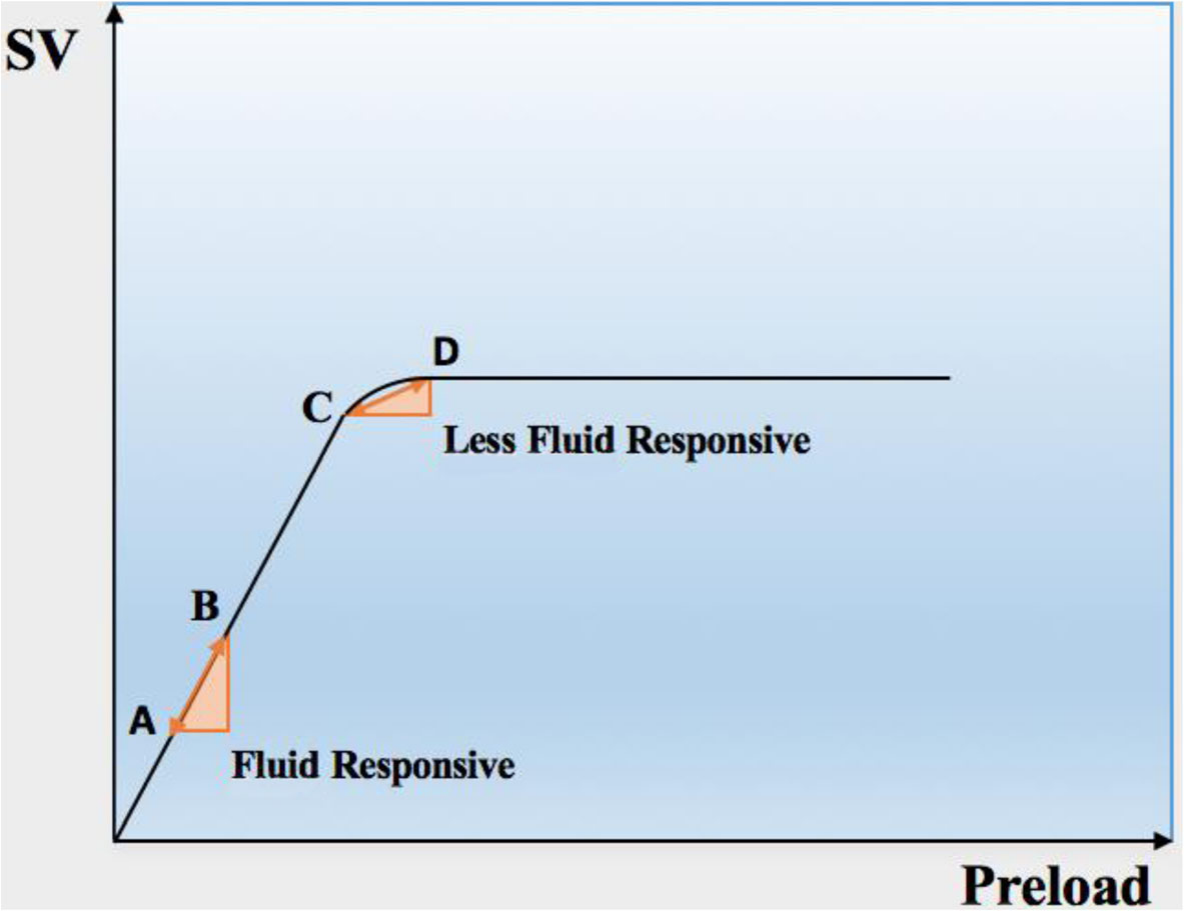
Frank–Starling curve of preload against left ventricular stroke volume (SV), demonstrating the change in SV induced by cyclic positive-pressure ventilation (A↔B and C↔D). The starting position on the curve indicates the level of variation possible in the SV after an increase in the preload. Once the patient ascends the curve (A → C), fluid responsiveness decreases in response to mechanical ventilation (C↔D)

The fundamentals underlying these parameters are based on simple physiology and are well represented in the Frank–Starling curve (Fig. 3). The cyclic positive-pressure ventilation intermittently increases pleural pressure and alters the filling conditions of the left and right ventricles. The increase in pleural pressure decreases venous return and right ventricle (RV) preload. Additionally, the RV afterload can increase due to an inspiratory rise in transpulmonary pressure. The decrease in the RV preload and the increase in the RV afterload decrease the RV SV, with minimum values reached end-inspiratory. The inspiratory reduction in the RV ejection will in a few beats decrease left ventricle (LV) preload and SV. The respiratory changes in the LV SV indicate a biventricular preload dependence and only occur in the ascending portion of the Frank–Starling curve [70].

Dynamic analyses of flow parameters need to be restricted to patients under controlled mechanical ventilation with tidal volumes between 8 and 10 ml/kg ideal body weight because heart–lung interactions are more complicated with spontaneous breathing efforts with or without a ventilator [71]. When right ventricular insufficiency is present, an experimental study of anesthetized pigs indicated that PPV and SVV only can be used to assess volume responsiveness during a volume-loading maneuver [72].

The use of PPV may be preferable to SPV and SVV because it is measured directly from the arterial pressure tracing and uses more advanced digital software [59]. Despite limitations in the use of dynamic indices and the inability to assess global ventricular function, SPV, PPV and SVV are currently the most precise predictors of fluid responsiveness [59], and their use does not require any specific training or the physical presence of the clinician during measurements.

In a recent study from Japan, SVV was compared with CVP and pulmonary artery diastolic pressure as estimates of the right and left ventricular preload in patients undergoing renal transplantation. As expected, SVV better predicted volume responsiveness [60]. A retrospective study from 2014 questioned whether SVV can be an alternative to CVP in kidney transplant recipients [73].

An overview of studies monitoring and targeting fluid therapy in kidney transplantation and their main outcomes is presented in Table 1. Most trials have been retrospective cohort studies. Errors due to bias and confounding variables are common and likely affect the results obtained using this study design. More robust clinical trials are necessary to better understand fluid management in kidney recipients.

**Table 1:** Monitoring and targeting of fluid therapy in kidney transplantation and the main outcomes

Reference	Year	Type of donor	Study design	Number of patients	Study group and aim	Main outcomes
Srivastava et al. [66]	2015	Living	Prospective nonrandomized control	110 Study, 104 control	Intraoperative fluid management TED-guided vs CVP-guided (historical controls)	Same rate of immediate graft functions in both groups. Less amount of fluid and less postoperative complications in TED-guided group
Aulakh et al. [27]	2015	Living	Retrospective	100	CVP > 12 mmHg vs CVP < 12 mmHg	Good early graft function if CVP = 12 mmHg
Aulakh et al. [27]	2015	Living	Retrospective	100	MAP > 100 mmHg vs MAP < 100 mmHg	Good early graft function if MAP > 95 mmHg
Toyoda et al. [60]	2015	Living	Prospective observational	31	SVV vs CVP vs DPAP as an estimate of RVEDVI in the same study group	SVV is a better indicator of preload
Chin et al. [73]	2014	No data	Retrospective	635	Ability of SVV to predict CVP in the same study group	SVV of 6% as an alternative to CVP of 8 mmHg
Gingell-Littlejohn et al. [28]	2013	No data	Observational	149	Effect of MAP on DGF	MAP < 70 mmHg associated with DGF
Campos et al. [4]	2012	No data	Retrospective	1966	Effect of MAP and CVP on graft function	Greater graft survival associated with MAP ≥ 93 mmHg. Perioperative fluid administration < 2500 ml associated with greater graft survival, whereas CVP ≥ 11 mmHg associated with high rates of ARE and chronic graft dysfunction
Bacchi et al. [37]	2010	Deceased	Observational	155	Correlation of CVP with DGF	CVP ≤ 8 mmHg correlates with DGF. Fluid input ≤ 2.25 L correlates with DGF
Othman et al. [7]	2010	Living	Randomized	40	Constant infusion rate of NaCl 0.9% at 10–12 ml/kg/h vs CVP at 5 mmHg during preischemia time. Post ischemia, the aim was CVP 8–10 mmHg in both groups	CVP target group had better graft function. Both groups received approximately 3 L of crystalloids. The CVP target group required fewer vasopressors and diuretics and had less postoperative tissue edema
Snoeijs et al. [5]	2007	Deceased (nonheart-beating)	Retrospective observational	177	Correlation of hemodynamic data with PNF of the graft	Average CVP < 6 mmHg and MAP < 110 mmHg were significant predictors of PNF. Preoperative diastolic BP < 80 mmHg was associated with PNF
Ferris et al. [30]	2003	Deceased and living	Retrospective	77	< 25% decline in CVP vs 25–50% decline in CVP vs > 50% decline in CVP in the immediate post-transplantation period	Neither absolute CVP nor % drop in CVP appeared to influence the rate on ATN. Reperfusion injury or related effects may be responsible for the CVP drop. No influence of volume of fluids infused on occurrence of ATN
Tóth et al. [11]	1998	Deceased	Prospective	121	Correlation of hemodynamic data with nonfunctioning grafts vs delayed graft function vs good graft function	Good graft function group had higher MAP (108 ± 26 mmHg)
Thomsen et al. [8]	1987	Deceased and living (51 vs 10)	Prospective nonrandomized control	61 (30 in group I, 31 in group II)	CVP not measured vs CVP kept > 5 cmH_2_O	Onset of graft function: Group I, 30%; Group II, 62%
Carlier et al. [10]	1982	Deceased	Prospective observational	120	Mean PAP ≤ 20 mmHg and diastolic PAP ≤ 15 mmHg vs mean PAP > 20 mmHg and DPAP > 15 mmHg	36% of ATN in Group I vs only 6% in Group II

*ARE* acute renal failure, *ATN* acute tubular necrosis, *BP* blood pressure, *CVP* central venous pressure, *DGF* delayed graft function, *DPAP* diastolic pulmonary artery pressure, *MAP* mean arterial pressure, *PNF* primary nonfunction, *PAP* pulmonary artery pressure, *RVEDVI* right ventricular end-diastolic pressure, *SVV* stroke volume variation, *TED* transesophageal Doppler

### Flow-directed fluid strategy: a more tailored approach than goal-directed fluid therapy?

Accumulating evidence supports the concept that fluid therapy should be individualized and based on dynamic indices of the intravascular volume. Conventional monitoring does not provide sufficient information for this approach, but novel technologies may allow titration of therapeutic interventions to ensure that oxygen delivery matches tissue need. One approach using dynamic indices has been goal-directed therapy (GDT). Goal-directed therapy decreased postoperative complications, hospital length of stay, mortality and hospital costs in high-risk surgical patients [17–19]. However, Pearse et al. [74] found no decrease in mortality after use of a CO-guided hemodynamic therapy algorithm compared with usual care. Goal-directed therapy protocols are often associated with an increased infusion volume, and often include significantly more colloids than crystalloids [75]. A factor that needs to be discussed in fluid management is not only the total amount of fluid infused but how, when and to which patient the volume is administered. There are no studies of GDT in kidney transplantation.

In contrast to GDT, which is guided by “goals”, flow-directed therapy dictates fluid administration based on “triggers” [62]. The triggers indicate that the clinical state has fallen below an acceptable level and may benefit from increased volume with an increase in CO. It does not aim for a higher than normal target, but rather is based on the clinical impression that the flow is inadequate for tissue needs and asks whether a given volume increased flow [62]. The change in CO is used as an indicator of the effectiveness of the therapy and not as the primary objective [62]. Thus, when the patient reaches the plateau of the Frank–Starling curve and the SV does not show a change after a fluid bolus, volume expansion is no longer the therapy of choice. Disregard for the status of fluid responsiveness before administering fluids can lead to the unnecessary delivery of fluids, even in GDT protocols. We advocate a flow-directed responsive protocol to be used in the management of perioperative fluid in kidney transplantation. Noninvasive CO monitoring is used to measure perfusion and assess fluid responsiveness when clinically indicated. After a volume is given (500 ml of crystalloid) the response of CO is checked. A 15% increase in CO with a CVP rise of at least 2 mmHg constitutes a positive response. When there is no appropriate response other therapies should be chosen (i.e., vasopressor and/or inotropic therapy to treat hypotension).

## Conclusions

By optimizing perioperative hemodynamic management in kidney transplantation, we hopefully can improve patient and kidney outcomes. The most recent studies of fluid therapy in the perioperative setting provide more questions than answers and challenge faith in traditional concepts. Effective perioperative fluid administration in kidney transplantation remains a black box, reinforced by the fact that fluid requirements are highly variable among patients and surgical procedures and the assessment of an individual patient’s intravascular volume remains challenging. It is unlikely that there will be a standardized all-inclusive algorithm, and a more highly individualized evidence-based approach adapted to each patient’s physiological needs and responses to volume and pharmacological therapy might be more helpful.

Prospective comparative clinical studies are warranted to better understand the use of dynamic analyses of flow parameters for optimal fluid management in specific patient populations such as kidney transplant patients, who often have impaired cardiovascular physiology and reduced hemodynamic autoregulation due to end-stage kidney disease.

## Abbreviations

AKI: Acute kidney injury
ATN: Acute tubular necrosis
BP: Blood pressure
CO: Cardiac output
CVP: Central venous pressure
DGF: Delayed graft function
GDT: Goal-directed therapy
HR: Heart rate
LV: Left ventricle
MAP: Mean arterial pressure
PAP: Pulmonary artery pressure
PPV: Pulse pressure variation
RV: Right ventricle
RVEDVI: Right ventricular end-diastolic volume index
SPV: Systolic pressure variation
SV: Stroke volume
SVV: Stroke volume variation
TED: Transesophageal Doppler
TEE: Transesophageal echocardiography
UOP: Urine output
